# Association between subconjunctival hemorrhage and hemorrhagic disorders: a nationwide population-based study

**DOI:** 10.1038/s41598-023-49428-z

**Published:** 2023-12-14

**Authors:** In Hwan Hong, Bum-Joo Cho, Se Hyun Choi

**Affiliations:** 1 Department of Ophthalmology, Hallym University College of Medicine, Chuncheon-si, Gangwon-do Republic of Korea; 2 Department of Ophthalmology, Hallym University Dongtan Sacred Heart Hospital, Hallym University Medical Center, Hwaseong-si, Gyeonggi-do Republic of Korea; 3 Department of Ophthalmology, Hallym University Sacred Heart Hospital, 22, Gwanpyeong-ro 170beon-gil, Dongan-gu, Anyang-si, Gyeonggi-do Republic of Korea; 4 Medical Artificial Intelligence Center, Hallym University Medical Center, Anyang-si, 14068 Republic of Korea

**Keywords:** Conjunctival diseases, Epidemiology

## Abstract

Subconjunctival hemorrhage (SCH) is a benign eye condition that is often noticeable and leads to medical attention. Despite previous studies investigating the relationship between SCH and cardiovascular diseases, the relationship between SCH and bleeding disorders remains controversial. In order to gain further insight into this association, a nationwide cohort study was conducted using data from the National Health Insurance Service-National Sample Cohort version 2.0 from 2006 to 2015. The study defined SCH using a diagnostic code and compared the incidence and risk factors of intracerebral hemorrhage (ICH) and gastrointestinal (GI) bleeding in 36,772 SCH individuals and 147,088 propensity score (PS)-matched controls without SCH. The results showed that SCH was associated with a lower risk of ICH (HR = 0.76, 95% CI = 0.622–0.894, *p* = 0.002) and GI bleeding (HR = 0.816, 95% CI = 0.690–0.965, *p* = 0.018) when compared to the PS-matched control group. This reduced risk was more pronounced in females and in the older age group (≥ 50 years), but not observed in males or younger age groups. In conclusion, SCH dose not increase the risk of ICH and major GI bleeding and is associated with a decreased incidence in females and individuals aged ≥ 50 years.

## Introduction

Subconjunctival hemorrhage (SCH) is a condition characterized by bleeding between the conjunctiva and episclera caused by the rupture of a subconjunctival blood vessel^1^. Although SCH is benign, self-limiting, and does not affect vision or cause pain, its noticeable appearance often leads to medical attention^2^. In fact, subconjunctival hemorrhage accounts for 3% of all patients who visit outpatient departments or emergency rooms for ophthalmic problems, especially 10.1% of the elderly over 65 years of age^3,4^. Patients visiting medical services for SCH are often concerned about underlying systemic conditions.

The causes of SCH are diverse, including traumatic and non-traumatic factors^5^. Traumatic causes are the major contributors to SCH, particularly among young patients, and often result from ocular injury, contact lens usage, ophthalmic surgery, or intravitreal injections^3,6–8^. On the other hand, non-traumatic causes of SCH are associated with various diseases such as conjunctivitis, tumors, inflammatory disorders, and diabetes, with hypertension having the strongest correlation, but the cause remains unknown in about 40% of cases^6,9–11^.

Since most patients discontinue treatment or testing without further investigation, it is difficult to establish a clear causal relationship due to the lack of prospective studies. However, recent advancements in big data research using nationwide population-based cohort studies have allowed for a small number of studies to be conducted on the association between cardiovascular diseases and SCH. These studies have shown that SCH was not significantly associated with the occurrence of ischemic vascular diseases such as acute coronary syndrome, acute myocardial infarction, and stroke^12,13^. Although a previous study had limitations in analyzing both ischemic stroke and intracerebral hemorrhage as one disease due to their different pathogenesis^12^, to the best of the authors' knowledge, no large-scale studies have been conducted on the association of hemorrhagic diseases other than ischemic diseases with SCH, and the topic remains controversial^14–18^. Therefore, the authors aim to investigate the association between SCH and intracerebral hemorrhage (ICH) and major gastrointestinal (GI) bleeding, a representative of major bleeding disorders.

## Results

**Figure 1:**
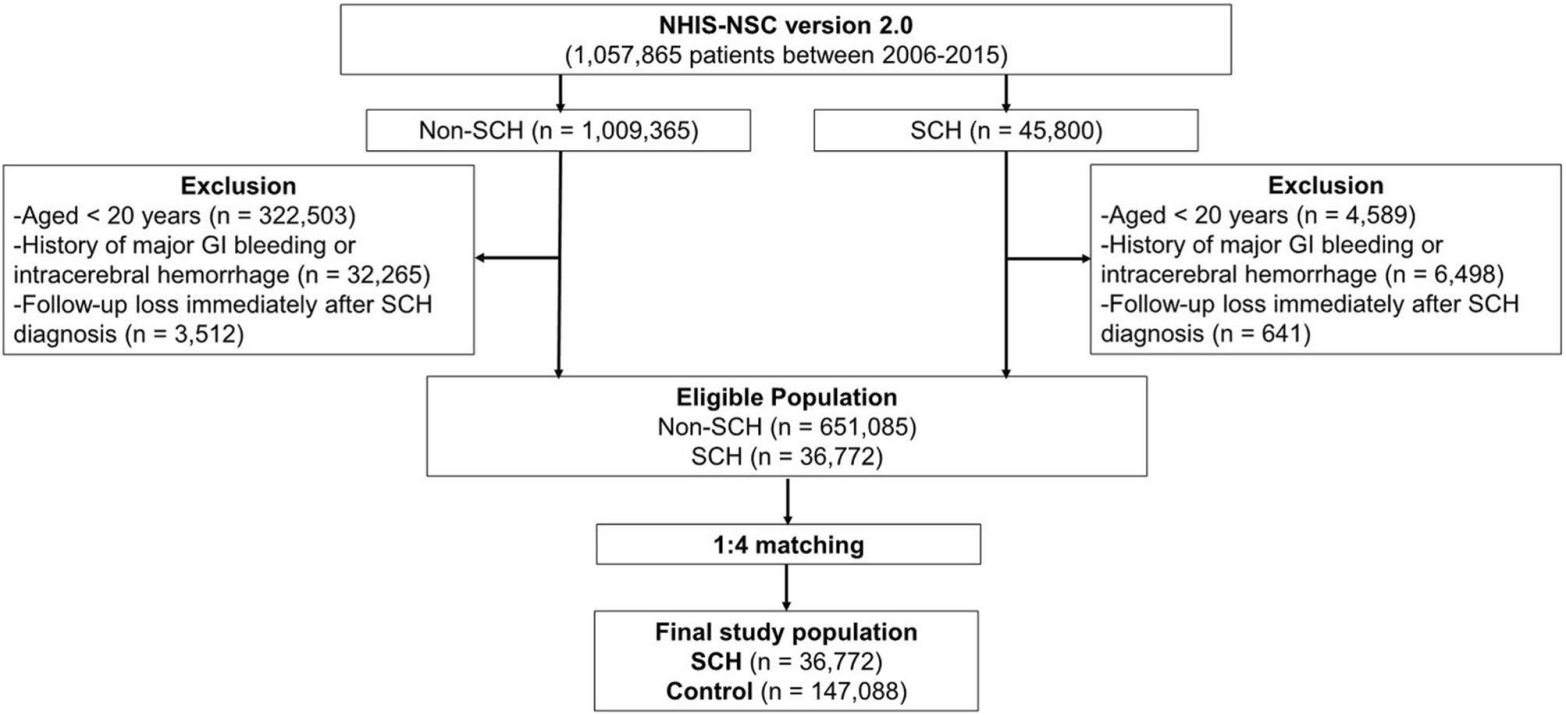
Schematic illustration of the participant selection process. This figure presents a schematic illustration of the selection process used to choose participants for the current study. Out of a total of 1,057,865 individuals, 36,772 patients with subconjunctival hemorrhage were paired with 147,088 control participants based on demographic factors such as age, sex, income group, region of residence, and prior medical history.

A total of 688,857 patients were eligible for the study (Fig. 1). The demographic characteristics of the eligible population are shown in Table 1. The SCH group tended to have a higher average age and a greater prevalence of chronic conditions compared to the non-SCH group, as indicated by the standardized mean difference (SMD) being greater than 0.1. However, after propensity score (PS)-matching (PSM), the age, gender, income level, region of residency, and chronic disease prevalence were comparable between the two groups (Table 2).

**Table 1:** General Characteristics of the Eligible Population Included in the Analysis.

Characteristics	SCH (n = 36,772)	Non-SCH (n = 651,085)	SMD
Age (years old)	51.4 ± 13.6	43.3 ± 15.6	0.548
Sex			0.069
Male	16,923 (46.0)	322,219 (49.5)	
Female	19,849 (54.0)	328,866 (50.5)	
Income			0.210
0 (lowest)	1306 (3.6)	29,285 (4.5)	
1	2685 (7.3)	57,564 (8.8)	
2	2455 (6.7)	44,882 (6.9)	
3	2351 (6.4)	46,799 (7.2)	
4	2625 (7.1)	61,887 (9.5)	
5	2805 (7.6)	59,492 (9.1)	
6	3113 (8.5)	54,751 (8.4)	
7	3757 (10.2)	78,301 (12.0)	
8	4118 (11.2)	63,776 (9.8)	
9	5241 (14.3)	76,230 (11.7)	
10 (highest)	6316 (17.2)	78,118 (12.0)	
Region of residence			0.044
Urban	18,089 (49.2)	305,826 (47.0)	
Rural	18,683 (50.8)	345,259 (53.0)	
Hypertension			0.396
Yes	11,950 (32.5)	103,286 (15.9)	
No	24,822 (67.5)	547,799 (84.1)	
Diabetes			0.374
Yes	9309 (25.3)	72,444 (11.1)	
No	27,463 (74.7)	578,641 (88.9)	
Dyslipidemia			0.618
Yes	14,031 (38.2)	81,235 (12.5)	
No	22,741 (61.8)	569,850 (87.5)	
Chronic kidney disease			0.100
Yes	430 (1.2)	2051 (0.3)	
No	36,342 (98.8)	649,034 (99.7)	
Chronic liver disease			0.277
Yes	5230 (14.2)	38,865 (6.0)	
No	31,542 (85.8)	612,220 (94.0)	
Ischemic heart disease			0.302
Yes	5454 (14.8)	37,472 (5.8)	
No	31,318 (85.2)	613,613 (94.2)	
Ischemic stroke			0.163
Yes	1807 (4.9)	12,701 (2.0)	
No	34,965 (95.1)	638,384 (98.0)	
Charlson Comorbidity Index (Mean ± SD)	3.31 ± 3.07	3.28 ± 3.09	0.011

SCH = subconjunctival hemorrhage. SMD = Standardized Mean Differences, Unless otherwise noted, values are n (%).

**Table 2:** General Characteristics of Patients with Subconjunctival Hemorrhage and Their Propensity Score-Based Matched Population Included in the Sensitivity Analysis.

Characteristics	SCH (n = 36,772)	Control (n = 147,088)	SMD
Age (years old)	51.4 ± 13.6	52.0 ± 14.8	0.046
Sex			0.031
Male	16,932 (46.0)	65,451 (44.5)	
Female	19,849 (54.0)	81,637 (55.5)	
Income			0.030
0 (lowest)	1306 (3.6)	4549 (3.1)	
1	2685 (7.3)	10,518 (7.2)	
2	2455 (6.7)	9623 (6.5)	
3	2351 (6.4)	9340 (6.3)	
4	2625 (7.1)	10,569 (7.2)	
5	2805 (7.6)	11,144 (7.6)	
6	3113 (8.5)	12,335 (8.4)	
7	3757 (10.2)	15,253 (10.4)	
8	4118 (11.2)	16,732 (11.4)	
9	5241 (14.3)	21,550 (14.7)	
10 (highest)	6316 (17.2)	25,475 (17.3)	
Region of residence			0.002
Urban	18,089 (49.2)	72,242 (49.1)	
Rural	18,683 (50.8)	74,846 (50.9)	
Hypertension			0.021
Yes	11,950 (32.5)	46,352 (31.5)	
No	24,822 (67.5)	100,736 (68.5)	
Diabetes			0.035
Yes	9309 (25.3)	35,027 (23.8)	
No	27,643 (74.7)	112,061 (76.2)	
Dyslipidemia			0.020
Yes	14,031 (38.2)	54,695 (37.2)	
No	22,741 (61.8)	92,393 (62.8)	
Chronic kidney disease			0.026
Yes	430 (1.2)	1333 (0.9)	
No	36,342 (98.8)	145,755 (99.1)	
Chronic liver disease			0.037
Yes	5230 (14.2)	19,039 (12.9)	
No	31,542 (85.8)	128,049 (87.1)	
Ischemic heart disease			0.036
Yes	5454 (14.8)	19,961 (13.6)	
No	31,318 (85.2)	127,127 (86.4)	
Ischemic stroke			0.020
Yes	1807 (4.9)	6599 (4.5)	
No	34,965 (95.1)	140,489 (95.5)	
Charlson Comorbidity Index (Mean ± SD)	3.23 ± 2.98	3.28 ± 3.09	0.015

SCH = subconjunctival hemorrhage. SMD = Standardized Mean Differences, Unless otherwise noted, values are n (%).

### Incidence of intracerebral hemorrhage and major gastrointestinal bleeding

**Figure 2:**
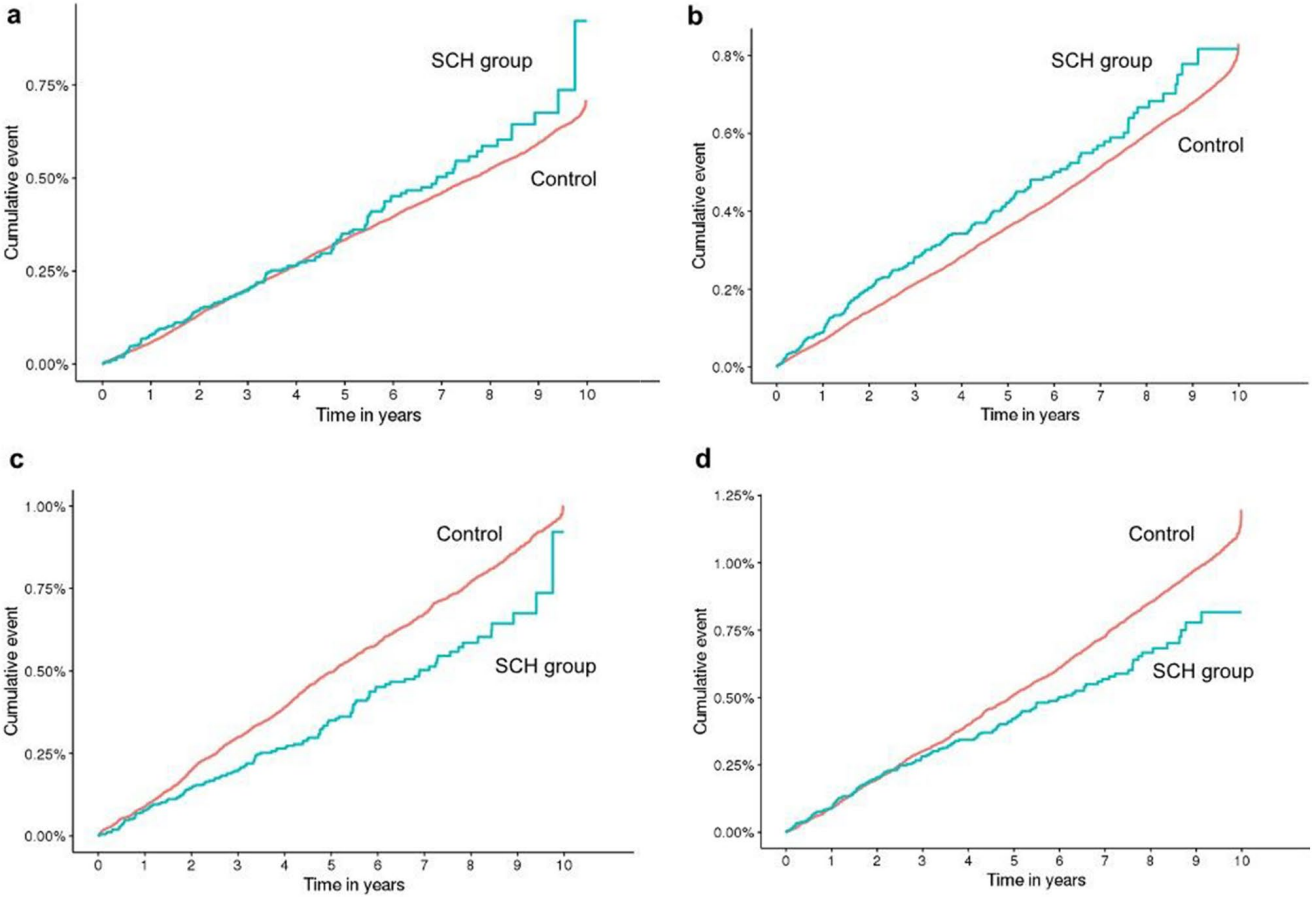
Cumulative Incidence of Intracerebral Hemorrhage (ICH) and Major Gastrointestinal (GI) Bleeding. This figure displays the cumulative incidence of ICH and major GI bleeding over time. Panels (**a**) and (**b**) illustrate the comparison between the subconjunctival hemorrhage (SCH) group and the general population, showing no significant difference in the incidence rates (*p* = 0.31 for ICH and *p* = 0.062 for GI bleeding, respectively). Panels (**c**) and (**d**) depict the comparison between the SCH group and the propensity score-matched (PS-matched) control group, where the SCH group exhibits a significantly lower incidence of both ICH (*p* = 0.0015) and major GI bleeding (*p* = 0.017). In all panels, the SCH group is represented by the blue solid line, and the control group is represented by the red line.

There was no statistically significant difference in the incidence of ICH or major GI bleeding between the SCH group and the general population (*p* = 0.31 and *p* = 0.062, respectively, log-rank test). However, the incidence of ICH and major GI bleeding was significantly lower in the SCH group compared to the PS-matched control group (*p* = 0.0015 and *p* = 0.017, respectively, log-rank test) (Fig. 2).

### Cox regression models for intracerebral hemorrhage and major gastrointestinal bleeding

Before PSM, there were 3,984 cases of ICH and 4,551 cases of major GI bleeding. The hazard ratios (HRs) of ICH (1.094, 95% Confidence Intervals [Cis] 0.919–1.302) and major GI bleeding (1.165, 95% CI 0.992–1.368) tended to be higher in the SCH group, but the difference was not statistically significant. After PSM, these ratios decreased in the SCH group (Table 3).

**Table 3:** Incidence Rate and Hazard Ratio of Intracerebral Hemorrhage and Major Gastrointestinal Bleeding after Subconjunctival Hemorrhage.

	Incidence rate (per 100 Person-Years)				
	SCH		Control		HR (95% CI) [*P*-value)
	Event	Incidence	Event	Incidence	SCH versus Control (ref)
Without propensity score matching					
ICH	132	0.199	3852	0.185	1.094 (0.919–1.302) [0.311]
Major GI bleeding	156	0.235	4395	0.211	1.165 (0.992–1.368) [0.062]
After propensity score matching					
ICH	132	0.199	1272	0.268	0.746 (0.622–0.894) [0.002]
Major GI bleeding	156	0.235	1438	0.303	0.816 (0.690–0.965) [0.018]

ICH = intracerebral hemorrhage, Major GI bleeding = major gastrointestinal bleeding, HR = hazard ratio, ref = reference.

### Exclusion of participants exposed to anticoagulants

To account for confounding factors, additional analyses were performed by excluding participants exposed to anticoagulants. Before PSM, the hazard ratios of ICH (1.083, 95% CI 0.905–1.297) and major GI bleeding (1.135, 95% CI 0.961–1.341) were slightly higher in the SCH group, but the difference was not statistically significant (*p* = 0.382 and *p* = 0.135, respectively). After PSM, the hazard ratios of ICH (0.748, 95% CI 0.62–0.902) and major GI bleeding (0.799, 95% CI 0.672–0.951) were lower in the SCH group (Supplementary Table S1). Furthermore, the incidence rate of ICH and major GI bleeding was significantly lower in the SCH group than in the control group after PSM (*p* = 0.0022 and *p* = 0.011, respectively, Supplementary Figure S1).

### Subgroup analysis by age and gender

After PSM, we observed a significant association between SCH and a lower incidence of ICH in female patients (HR: 0.666, 95% CI 0.512–0.866, *p* = 0.002). This association was not observed in male patients (HR: 0.832, 95% CI 0.647–1.07, *p* = 0.152). Similarly, the risk of major GI bleeding showed a significant reduction in female patients (HR: 0.705, 95% CI 0.523–0.950, *p* = 0.022), but not in male patients (HR: 0.856, 95% CI 0.699–1.049, *p* = 0.134). Among patients over 50 years of age, there was a significant association between SCH and reduced incidences of both ICH (HR: 0.697, 95% CI 0.569–0.855, *p* < 0.001) and GI bleeding (HR: 0.747, 95% CI 0.613–0.911, *p* = 0.004). However, in patients younger than 50 years, this association was not statistically significant (*p* = 0.743 for ICH and *p* = 0.58 for GI bleeding) (Supplementary Table S2).

## Discussion

The study analyzed 688,857 patients to examine the association between subconjunctival hemorrhage and the incidence of ICH and major GI bleeding. The results showed that patients with SCH tended to be older and have more chronic diseases compared to those without SCH. After PSM, the age, sex, income level, region of residence, and prevalence of chronic diseases were comparable between the two groups.

The results of the study showed that the incidence of ICH and major GI bleeding was significantly lower in the SCH group when compared to the PSM control group, but there was no statistical difference when compared to the general population. The hazard ratios of ICH and major GI bleeding tended to be higher in the SCH group before PSM, but after PSM, the hazard ratios were lower in the SCH group.

Previous studies have shown that SCH can be associated with various systemic diseases, including hypertension, diabetes mellitus, and cardiovascular disease^3,5,13^. Our findings are consistent with these studies, as we observed a higher prevalence of chronic diseases in the SCH group compared to the non-SCH group before PSM. Several previous studies have suggested that SCH may be a sign of underlying systemic diseases, including hypertension, diabetes, and hematological disorders^9,13^. These conditions can cause increased vascular fragility, leading to the development of SCH. This association has also been shown for other ocular hemorrhages, such as vitreous hemorrhage and retinal hemorrhage, which have been linked to systemic diseases, such as diabetes and hypertension^19–23^.

However, after accounting for these confounding factors in PSM, SCH were association with lower incidences of ICH and GI bleeding. This could be due to comprehensive medical management, including the use of antiplatelet agents to prevent coronary heart disease and stroke, and medications for treating chronic diseases that might cause adverse GI reactions.

Additionally, our subgroup analysis indicated a lower observed risk of ICH and GI bleeding in patients with SCH, particularly in females and in those aged 50 years or older. This suggests that SCH could be more than a benign ocular issue, potentially acting as an indicator of systemic health status, especially in certain demographic groups. One possible reason for the difference in the lower prevalence of major bleeding between young men and elderly women is due to the underlying cause of SCH. It is believed that young men tend to develop SCH mainly due to trauma, whereas in elderly women, SCH may be a sign of underlying systemic diseases such as hypertension, which are known risk factors for both SCH and ICH and major GI bleeding. Hormonal differences in females may also play a role, as previous studies have indicated that estrogen might have protective effects on the cardiovascular system^24,25^. It is also established that estrogen and progesterone can influence ocular structures, causing congestion, hyperemia, and secondary bleeding by increasing capillary permeability^26,27^. These findings warrant further investigation in diverse populations and settings to assess their generalizability and to fully understand the implications of these hormonal effects.

The study results should be interpreted with consideration of the following limitations. Firstly, the limitation of this study is the use of a retrospective database. Although the National Health Insurance Service-National Sample Cohort (NHIS-NSC) version 2.0 is a large, representative sample of the Korean population, there may still be some inaccuracies in the collected data, such as incomplete or missing information. Secondly, the study used diagnoses from the International Classification of Diseases-10th edition, which can result in coding errors or inconsistencies. Thirdly, our subgroup analysis involved the exclusion of patients who were exposed to anticoagulants. While this was intended to reduce confounding factors, it may have introduced a selection bias. Additionally, we did not evaluate the use of antiplatelet agents and NSAIDs, which are recognized for their association with a higher incidence of SCH, thus presenting a limitation in our analysis^17,28^. Fourthly, this study lacks information on lifestyle factors, such as smoking, alcohol consumption, and physical activity, which can impact the development of SCH and the primary outcome events of GI bleeding and ICH. This may result in residual confounding that cannot be fully adjusted for using the methods used in this study. Finally, this study was conducted in Republic of Korea and may not be generalizable to other populations. Further studies in different populations and settings are needed to validate these findings.

## Conclusions

In conclusion, this study analyzed the association between subconjunctival hemorrhage and the incidence of intracerebral hemorrhage and major GI bleeding in 688,857 patients. The results showed that patients with SCH tended to have more chronic diseases compared to those without SCH. However, after propensity score matching, the confounding factors were eliminated and the incidence of intracerebral hemorrhage and major GI bleeding was found to be significantly lower in the SCH group compared to the matched control group. Furthermore, subgroup analysis based on age and sex showed that SCH was significantly associated with a decrease in the incidence of intracerebral hemorrhage and major GI bleeding in females and individuals aged ≥ 50 years.

Our findings suggest that SCH, often perceived as a benign and isolated ocular condition, may signal broader health concerns in certain patient groups. It is important to note, however, that our study establishes an associative link rather than causality. This necessitates further investigation, especially through prospective studies, to understand the underlying mechanisms of this association. Future research should delve into the biological factors underlying these associations, such as hormonal differences or vascular integrity, and consider potential confounders like medication use, lifestyle factors, and comorbid conditions. Additionally, while our study offers valuable insights from a large population-based cohort, exploring these associations in diverse populations and settings is crucial. This would help determine the generalizability of our findings and further elucidate the role of SCH as a potential marker for systemic vascular conditions.

## Materials and methods

### Study design and data collection

The study was approved by the Institutional Review Board of Hallym University (IRB No. 2020–07-001) and was conducted in accordance with the Declaration of Helsinki and Hallym University's guidelines. The Institutional Review Board of Hallym waived the need for written informed consent since the personal IDs of the Korean Health Insurance Review and Assessment Service-National Sample Cohort (HIRA-NSC) were scrambled.

The research utilized data from the Korean HIRA-NSC, which has been established by the National Health Insurance Service (NHIS) in the Republic of Korea. The NHIS has been gathering health insurance records from healthcare providers and the sample used in this study was extracted from 1,108,369 individuals who maintained their health insurance status in 2006, representing approximately 2% of the national population. For these subjects, information was collected retrospectively from 2002 to 2005 and prospectively from 2006 to 2015. The participants of the NHIS-NSC 2.0 were chosen to represent the Korean population using 1476 constructed strata including age group, sex, and income level. The NHIS-NSC 2.0 has five databases: birth and death, insurance eligibility, treatment (records of healthcare service, expense, and medication), general health examination, and clinic (information on healthcare providers) for the period 2002–2015. The diagnoses were coded using the International Classification of Diseases-10th edition. A detailed description of the data source can be found in previous studies^29–32^.

### Study populations and cohort definitions

We identified patients who were diagnosed with SCH between January 2006 and December 2015 (diagnostic code: H11.3). We excluded patients aged < 20 years; those with a history of major GI bleeding or ICH, those who lost follow-up immediately after the index date. Histories of major GI bleeding (diagnostic code: K26.0, K26.2, K26.4, K26.6, K27.0, K27.2, K27.4, K27.6, K28.0, K28.2, K28.4, K28.6, K29.0, K62.5, K92.0, K92.1, K92.2), and ICH (diagnostic code: I60-62) were identified using ICD-10 codes. These methods were used in other studies that evaluated the incidence of cardiovascular disease in Korea^33–35^.

The SCH subjects were matched 1:4 with the subjects among this cohort who were never diagnosed with SCH from 2006 to 2015 (the control group). The matches were processed by age, sex, income group, region of residence, and prior medical history (hypertension, diabetes, dyslipidemia, chronic kidney disease, chronic liver disease, ischemic heart disease, and ischemic stroke). To prevent selection bias when choosing matched subjects, the control group subjects were sorted using a random number order and were then selected from top to bottom. It was assumed that each patient with SCH and the matching control subjects were receiving any needed medical treatment during concurrent time periods. Therefore, the control group subjects who died before the index date were excluded. In both the SCH and control groups, the subjects with a history of major GI bleeding or ICH prior to the index date were excluded from this study. Finally, 1:4 matching resulted in the inclusion of 36,772 SCH patients and 654,597 control participants (Fig. 1).

### Statistical analysis

In this study, discrete or categorical variables are presented as numbers and percentages, and continuous variables as means with standard deviations or medians. The incidence of clinical events was calculated using the Kaplan–Meier estimate and compared through a log-rank test. A multivariable Cox proportional hazards regression was applied to determine the adjusted HRs and 95% confidence intervals CIs for comparing the risk of GI bleeding and ICH after index SCH. To identify the independent predictors of the primary outcome, the multivariable Cox proportional hazards model was employed, and C-statistics and 95% CIs were calculated to evaluate the discriminant function of the model. For subgroup analyses, the participants were divided into two age groups (20–49 and 50 + years) and two sexes (male and female).

Inverse probability weighting (IPW) Cox proportional hazards regression was utilized to reduce the confounding effect. The PS was estimated through a multivariable logistic regression model, which included all variables. PS-matched cohort analysis was used to compare the differences in clinical outcomes between groups. The PSM was done by selecting the closest match in a 1:4 ratio without replacements. The balance of covariates between the two groups after PS matching was evaluated by computing SMDs, and an SMD of less than 0.1 was considered well-balanced.

All analyses were two-tailed, and a *p*-value less than 0.05 was considered statistically significant. The statistical analyses were performed using R version 3.3.3 (R Foundation for Statistical Computing).

### Supplementary Information

Supplementary Figure S1.

Supplementary Table S1.

Supplementary Table S2.

## Data Availability

Data can be made available by the corresponding author upon reasonable request.
