# Supplementary figures and images for: Association between subconjunctival hemorrhage and hemorrhagic disorders: a nationwide population-based study
Source: Sci Rep. 2023 Dec 14;13:22237. doi: 10.1038/s41598-023-49428-z (PMC10721604; doi:10.1038/s41598-023-49428-z)

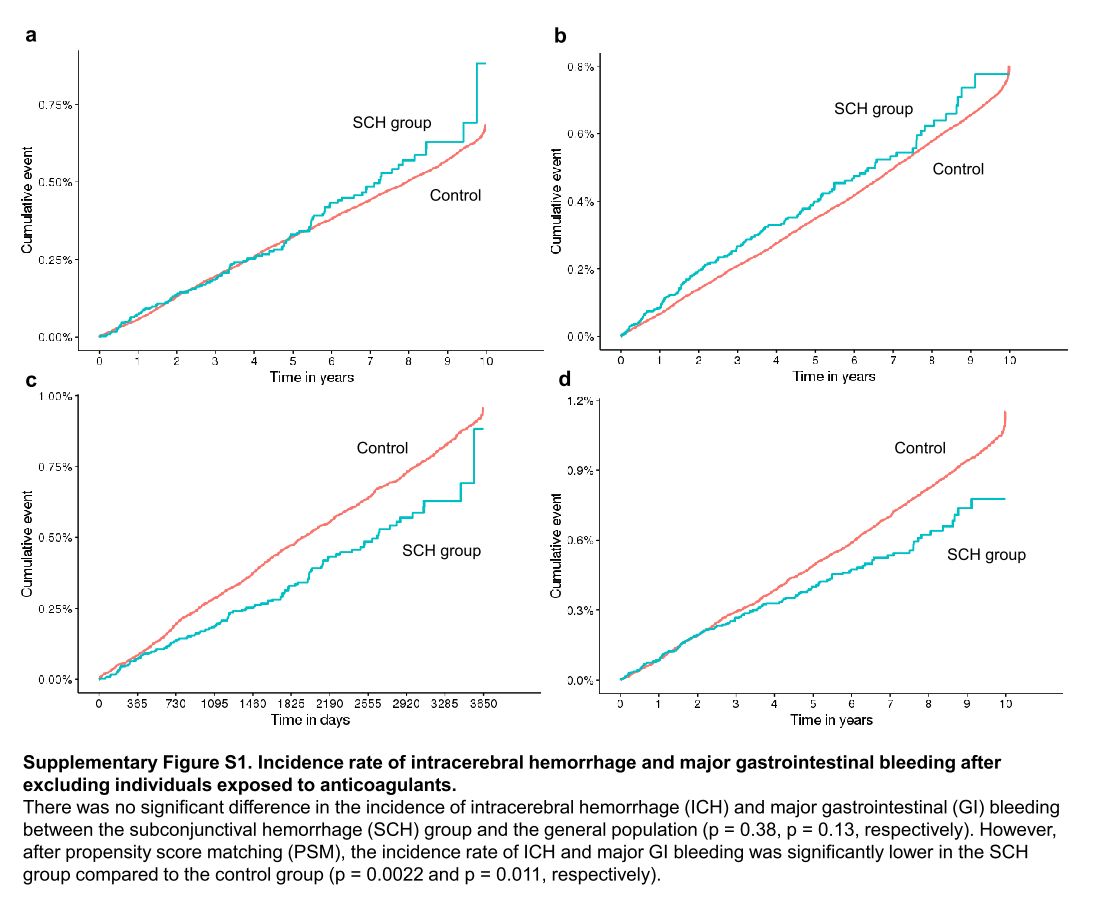

Supplement: Supplementary file 1 — Supplementary Figure S1. [file 41598_2023_49428_MOESM1_ESM.jpg]
