# Supplementary material for: Association between subconjunctival hemorrhage and hemorrhagic disorders: a nationwide population-based study
Source: Sci Rep. 2023 Dec 14;13:22237. doi: 10.1038/s41598-023-49428-z (PMC10721604; doi:10.1038/s41598-023-49428-z)
Supplement: Supplementary file 2 — Supplementary Table S1. [file 41598_2023_49428_MOESM2_ESM.docx]

**Supplementary Table S1. Incidence Rate and Hazard Ratio of Intracerebral Hemorrhage and Major Gastrointestinal Bleeding after Subconjunctival Hemorrhage (Exclusion of Participants Exposed to Anticoagulants)**

|  | Incidence rate (per 100 Person-Years) | | | | |
| --- | --- | --- | --- | --- | --- |
|  | SCH | | Control | | HR (95% CI) [P-value] |
|  | Event | Incidence | Event | Incidence | SCH vs Control (ref) |
| Without propensity score matching | | | |  |  |
| ICH | 124 | 0.19 | 3681 | 0.178 | 1.083 (0.905-1.297) [0.382] |
| GI bleeding | 145 | 0.222 | 4206 | 0.204 | 1.135 (0.961-1.341) [0.135] |
| After propensity score matching | | | |  |  |
| ICH | 124 | 0.19 | 1194 | 0.256 | 0.748 (0.62-0.902) [0.002] |
| GI bleeding | 145 | 0.222 | 1361 | 0.292 | 0.799 (0.672-0.951) [0.011] |

ICH = intracerebral hemorrhage, GI bleeding = gastrointestinal bleeding, HR = hazard ratio, ref = reference.
