# Supplementary material for: Association between subconjunctival hemorrhage and hemorrhagic disorders: a nationwide population-based study
Source: Sci Rep. 2023 Dec 14;13:22237. doi: 10.1038/s41598-023-49428-z (PMC10721604; doi:10.1038/s41598-023-49428-z)
Supplement: Supplementary file 3 — Supplementary Table S2. [file 41598_2023_49428_MOESM3_ESM.docx]

**Supplementary Table S2. Subgroup analysis of hazard ratio of subconjunctival hemorrhage for intracerebral hemorrhage and major gastrointestinal bleeding according to sex and age**

|  | ICH | | | | GI bleeding | | | |
| --- | --- | --- | --- | --- | --- | --- | --- | --- |
|  | Before matching | | After matching | | Before matching | | After matching | |
|  | HR (95% CI) | P-value | HR (95% CI) | P-value | HR (95% CI) | P-value | HR (95% CI) | P-value |
| Male |  |  |  |  |  |  |  |  |
| SCH | 1.193 (0.939-1.516) | 0.148 | 0.832 (0.647-1.07) | 0.152 | 1.288 (1.062-1.562) | 0.010 | 0.856 (0.699-1.049) | 0.134 |
| Control | 1 |  | 1 |  | 1 |  | 1 |  |
| Female |  |  |  |  |  |  |  |  |
| SCH | 1.006 (0.78-1.297) | 0.962 | 0.666 (0.512-0.866) | 0.002 | 1.011 (0.757-1.349) | 0.943 | 0.705 (0.523-0.950) | 0.022 |
| Control | 1 |  | 1 |  | 1 |  | 1 |  |
| Old Age (Age≥ 50) |  |  |  |  |  |  |  |  |
| SCH | 0.676 (0.556-0.823) | < 0.001 | 0.697 (0.569-0.855) | < 0.001 | 0.784 (0.647-0.949) | 0.013 | 0.747 (0.613-0.911) | 0.004 |
| Control | 1 |  | 1 |  | 1 |  | 1 |  |
| Young Age (Age < 50) |  |  |  |  |  |  |  |  |
| SCH | 1.284 (0.882-1.871) | 0.192 | 1.069 (0.716-1.597) | 0.743 | 1.286 (0.959-1.726) | 0.093 | 1.093 (0.798-1.497) | 0.580 |
| Control | 1 |  | 1 |  | 1 |  | 1 |  |

ICH = intracerebral hemorrhage, GI bleeding = gastrointestinal bleeding, HR = hazard ratio, ref = reference.
